# Genetic Parameters and Genomic Regions Underlying Growth and Linear Type Traits in Akkaraman Sheep

**DOI:** 10.3390/genes13081414

**Published:** 2022-08-10

**Authors:** Mehmet Kizilaslan, Yunus Arzik, Stephen N. White, Lindsay M. W. Piel, Mehmet Ulas Cinar

**Affiliations:** 1 Department of Animal Science, Faculty of Agriculture, Erciyes University, Kayseri 38039, Turkey; 2 International Center for Livestock Research and Training Center, Ministry of Agriculture and Forestry, Ankara 06852, Turkey; 3 Department of Veterinary Microbiology and Pathology, College of Veterinary Medicine, Washington State University, Pullman, WA 99164, USA; 4 USDA-ARS Animal Disease Research, Washington State University Pullman, Pullman, WA 991646630, USA

**Keywords:** genetic parameters, QTL, GWAS, growth, linear type traits, sheep

## Abstract

In the current study, the genetic architecture of growth and linear type traits were investigated in Akkaraman sheep. Estimations of genomic heritability, genetic correlations, and phenotypic correlations were implemented for 17 growth and linear type traits of 473 Akkaraman lambs by the univariate and multivariate analysis of animal mixed models. Correspondingly, moderate heritability estimates, as well as high and positive genetic/phenotypic correlations were found between growth and type traits. On the other hand, 2 genome-wide and 19 chromosome-wide significant single nucleotide polymorphisms were found to be associated with the traits as a result of animal mixed model-based genome-wide association analyses. Accordingly, we propose several genes located on different chromosomes (e.g., *PRDM2, PTGDR, PTPRG, KCND2, ZNF260, CPE, GRID2, SCD5, SPIDR, ZNF407, HCN3, TMEM50A, FKBP1A, TLE4, SP1, SLC44A1*, and *MYOM3*) as putative quantitative trait loci for the 22 growth and linear type traits studied. In our study, specific genes (e.g., *TLE4, PTGDR,* and *SCD5*) were found common between the traits studied, suggesting an interplay between the genetic backgrounds of these traits. The fact that four of the proposed genes (*TLE4, MYOM3, SLC44A1,* and *TMEM50A*) are located on sheep chromosome 2 confirms the importance of these genomic regions for growth and morphological structure in sheep. The results of our study are therefore of great importance for the development of efficient selection indices and marker-assisted selection programs, as well as for the understanding of the genetic architecture of growth and linear traits in sheep.

## 1. Introduction

Sheep (*Ovis aries*) are among the most utilized animals since domestication. The hardy and adaptive nature of the sheep allowed the species to be widely distributed along various environmental settings and diverse terrains, including arid areas, which promoted the diversification of the breeds. Additionally, the variety of resources provided by sheep established the species as an essential component of the global agricultural economy. Indigenous sheep breeds, especially, have a wide range of adaptive traits that have evolved for thousands of generations with natural and artificial selection, which constitute the main pillars of food security and sustainable production in many countries. Likewise, Akkaraman (i.e., White Karaman) sheep, a widely raised fat-tailed sheep breed of Türkiye, is known for its hardy nature, capable of surviving and reproducing under extreme conditions [1,2]. As a dual-purpose breed, the Akkaraman has quite a large share of Türkiye’s sheep population with considerably low meat productivity as indicated by slaughtered lamb weight [1]. Economically, sheep and lamb meat holds the fourth biggest share of global meat production [3]. Therefore, traits correlated with meat production, such as growth and linear type traits, which are mainly composed of body condition score, chest and rump width, body length, feet angle, and leg posture traits, have great importance in terms of breeding practices for high-yielding, prime lambs. In fact, both trait groups have a considerably high influence on important economic traits such as productivity, disease resistance, mobility, reproductive performance, and certain physiological and biochemical parameters [4,5]. Therefore, improving the selection accuracy for these traits would enhance the overall efficiency and profitability of a sheep production system.

Although linear type traits have been around for dairy cattle, goats, and dairy sheep for decades, studies conducted on meat sheep are very limited [6,7,8]. Accordingly, several scales were proposed for scoring meat sheep for type traits, such as those based on 1 to 5 and 1 to 9 scales [5,9,10,11]. Studies concerning genetic parameters and the genetic background of growth and linear type traits in sheep are quite scarce [5,10,12]. It is widely known that growth and linear type traits in sheep are commonly affected by environmental factors such as nutrition and seasonal variation, as well as the animals’ genetic background. In fact, moderate heritabilities and a wide range of genetic correlations were found for growth and linear type traits within various sheep breeds, which indicates a great potential for utilizing the genetic determination behind those complex traits to achieve faster genetic improvement [5,9,13,14,15]. A comprehensive pedigree is required, where countries significantly lack pedigree and trait recording. To overcome this problem, it was proposed that genomic information be exploited through the use of a genomic relationship matrix [16,17]. Additionally, using genomic information has been proven to increase the selection accuracy of genetic improvement programs and positively affect the acquired genetic gain [18,19]. A major prerequisite for implementing marker- and gene-assisted selection for economically important traits such as growth performance and linear type traits is the identification of the relevant candidate genes and quantitative trait loci (QTL). Unfortunately, sheep have a relatively small number of discovered QTLs compared to those concerning cattle. Among the present sheep QTLs, studies aiming at the discovery of QTLs associated with growth and linear type traits are very few [20].

The massive advancement of next-generation sequencing technologies has resulted in a wide range of high-throughput genotyping methods. With the availability of high-density Single Nucleotide Polymorphism (SNP) arrays, a major opportunity for the implementation of the genome-wide association studies (GWAS) to reveal the QTL underlying traits of economic interest in livestock production has been provided [21]. GWA studies have proven to be a powerful tool to identify genetic variants and candidate genes associated with a wide range of traits in a wide range of farm animals including cattle, goats, and sheep [8,12,22]. Up until recent years, only a few GWASs were conducted on certain growth [12,23,24,25,26] and linear type traits [10] of sheep, which have suggested putative QTLs on OAR1, 2, 3, 6, 8, 9, 14, 15, 16, 17, 19, and 22 for growth traits and OAR2, 5, 16, 23, and 26 for various linear type traits.

However, the scarcity of discovered QTLs, the low overlap among identified loci, and the complex nature of the genetic background of growth and linear type traits emphasize the need for a deep understanding of the genetic architecture of those traits as well as the need for more QTL discovery studies in sheep. Therefore, the main objectives of the present study were to estimate the genomic heritabilities, observe correlations for certain growth and linear type traits of Akkaraman sheep, and implement GWASs to understand the genetic mechanisms behind complex traits with potential implications in the genetic improvement of an indigenous sheep breed.

## 2. Materials and Methods

### 2.1. Animals and Phenotyping

The authors followed the suggestions by the ARRIVE guidelines throughout the study [27]. The experimental population consisted of 473 randomly selected Akkaraman lambs (i.e., 186 male and 287 female lambs) sourced from three different large-scale commercial farms located near the outskirts of the Ankara province. The climate of the studied region is characterized by harsh and cold winters, while summers are extremely hot and dry. This results in a meager, poor-quality grassland diet for grazing animals. Since the animals were registered to the National Community-based Small Ruminant Breeding Programme, a transgenerational phenotypic selection is applied to the ancestors of the studied animals. Within each generation, the best performing rams are mated to the best performing does, where performance is measured in growth rate. Lambs were born between January and February 2021 and weaned between April and May 2021, with an average weaning age of 3 months. Once the lambs were weaned, 372 lambs were kept on grazing pasture until 6 months of age while 101 lambs were finished for a period of 90 days with 750–1000 g concentrate feed (i.e., a proportional mixture of barley, corn, and wheat) per day as well as hay.

Birth weight (BW), weaning weight (WW), and 180 days weight (180DW) records were taken from the lambs. From those, pre-weaning average daily gain (preADG), post-weaning average daily gain (postADG), and 180 days average daily gain (180ADG) were derived. Weaning weight here is the adjusted weight of the lambs at 90 days, while 6 months weight is the adjusted weight at 180 days via linear interpolation. Additionally, when the lambs were at an average of 6 months old, scoring for 11 type traits including body condition score (BCS), tail size (TS), rear legs rear view (RLRV), gigot roundness rear view (GRRV), rump width (RW), rear legs width (RLW), rear legs feet angle (RLFA), gigot roundness side view (GRSV), rear legs side view (RLSV), body length (BL), and chest width (CW) were implemented following also the descriptions provided by [5,9,10,11]. Blood samples were collected right after the scoring, where approximately 6 mL of blood samples were taken from *Vena jugularis* into an EDTA-coated vacutainer. Samples were immediately transferred within a cold chain to the Genetics Laboratory of the International Center for Livestock Research and Training, Ankara, Turkiye, where the DNA extraction and genotyping were executed.

The recorded environmental factors to be accounted for as fixed effects were sex (two levels), birth type (two levels), herd (three levels), and feed type (two levels). The preliminary statistics, data pruning, and model fitting were implemented via the R statistical environment. Continuous data were checked for normality via the Shapiro–Wilk test, where outliers with observations deviating three standard deviations ± mean for each trait were excluded from further analyses. Furthermore, the heteroscedasticity of variances was tested with the Breusch–Pagan test [28]. The descriptive statistics of the phenotypic observations after the outliers were removed and the quality control performed are provided in Supplementary Table S1.

### 2.2. Genotyping and Quality Control

DNA was extracted from those collected blood samples with the QIAcube HT automated device using a commercial Blood/Tissue DNA purification kit and the relevant manufacturer’s protocol provided by the same company (Qiagen, Hilden, Germany). The purified DNA was exposed to a quality/quantity check step to ensure that all samples passed the minimum genotyping requirements of A_260/280_ > 1.8; A_260/230_ > 1.5 and a sample amount of >20 ng/µL with a MultiSkan SkyHigh UV/VIS microplate spectrophotometer (ThermoFisher Scientific, Waltham, MA, USA). After checking for DNA integrity on gel electrophoresis, the samples proceeded to genotyping, which was implemented using the Axiom™ Ovine SNP Genotyping Array (50 K) and the GeneTitan™ Multi-Channel Instrument according to the manufacturer’s guide (Axiom™ 2.0 Assay 96-Array Format Manual Workflow, ThermoFisher Scientific, Waltham, MA, USA).

Preceding further analyses, a quality control (QC) step was performed to ensure lower Type-I and Type-II error rates [29,30,31]. Correspondingly, the ‘GenABEL’ R package [32] was used to remove SNPs with a minor allele frequency (MAF) <0.05, call rate <95%, and those that are mapped to sex chromosomes and deviate from the Hardy–Weinberg equilibrium (*p*-value cut-off = 0.05/number of SNPs). Furthermore, samples with an individual call rate below 90%, identity by state (IBS) of >95%, and those with too high heterozygosity (False Discovery Rate < 1%) were also removed from the data. This resulted in 40,439 remaining SNPs passing imposed QC criteria for follow-up statistical analyses.

### 2.3. Genomic Heritability and Correlations

Genomic heritability estimates and genetic correlations were obtained using univariate and bivariate analyses of the linear animal mixed model, which is given below with the variance-covariance structure of the estimations, on ‘sommer’ R package [33]:

$$y=X\beta +Zu+e\;V=\left[\begin{array}{ccc} Z_{i}G\sigma_{u_{i}}^{2}Z_{i}^{\prime }+I\sigma_{e_{i}}^{2} & \cdots & Z_{i}G\sigma_{u_{i,j}}Z_{j}^{\prime }+I\sigma_{e_{i,j}}\\ \vdots & \ddots & \vdots \\ Z_{i}G\sigma_{u_{i,j}}Z_{j}^{\prime }+I\sigma_{e_{i,j}} & \cdots & Z_{j}G\sigma_{u_{j}}^{2}Z_{j}^{\prime }+I\sigma_{e_{j}}^{2} \end{array}\right]$$

Here, **y** refers to the vector containing phenotypic observations, β is the vector of fixed effects included in the model, **u** is the vector of random effects for breeding values, where u~MVN (0, **G**$\sigma_{u}^{2}$), **e** is the vector of random residual effects, assumed to be from e~MVN (0, **I**$\sigma_{e}^{2}$), and **X** and **Z** are the design matrices that are, respectively, mapping fixed environmental effects and random breeding values to the observations. Additionally, $\sigma_{u}^{2}$ and $\sigma_{e}^{2}$ are the additive genetic variance and random residual variances for the trait of interest, where ‘i’ and ‘j’ represent different traits to be analyzed for bivariate analyses, and **I** is the identity matrix. Below, **G** is the genomic relationship matrix obtained by the method proposed by [17]:

$$G=\frac{ZZ^{\prime }}{2\sum p_{i}\left(1-p_{i}\right)}$$

where, when divided by $2\sum p_{i}\left(1-p_{i}\right)$, the G matrix becomes similar to that of a numerator relationship matrix which was obtained from a pedigree.

Heritability estimates here are the narrow sense heritability ($h^{2}=\frac{\sigma_{u}^{2}}{\sigma_{p}^{2}}$) where $\sigma_{u}^{2}$ is the additive genetic variance and $\sigma_{p}^{2}$ is the total phenotypic variance (i.e., composed of $\sigma_{u}^{2}+\sigma_{e}^{2}$) obtained from the univariate analysis of the trait of interest with the general model above. The components of (co)variance were obtained by the Newton–Raphson optimization method using a direct inversion (DI)-based restricted maximum likelihood (REML) approach to solve the mixed model above, using the genomic relationship matrix constructed from the IBS information provided by the SNP markers [34,35]. Heritability estimates and genetic and phenotypic correlations are given in Supplementary Table S2, where diagonal values represent the heritability estimate of the relevant trait, above the diagonal is the phenotypic correlation, and below the diagonal is the genetic correlation for the matching traits. Fixed effects were omitted during the bivariate analyses for obtaining genetic correlations to avoid any possible convergence problems. Standard errors of the genetic correlations were estimated by using a second order Taylor series expansion on the delta method as explained by [36].

### 2.4. Genome-Wide Association Analysis

Univariate genome-wide association analysis for each trait was carried out using the linear mixed model presented above, which employs a procedure on the ‘GenABEL’ R package originally proposed by Chen and Abecasis [37]. For this purpose, the genomic relationship matrix was also used to account for any possible covariance between the trait measurements of animals that is attributable to population stratification and cryptic relatedness, which would lead to an increased Type I error rate [38,39]. Briefly, the two-step approach for solving the mixed model equation above involves an initial step of accounting for all fixed effects other than SNP effects (i.e., the recorded environmental factors) and estimating the (co)variance components, where the residuals of this primary analysis are later used to estimate the additive genetic effects of SNPs which are fitted as fixed factors one at a time.

Quantile–Quantile (Q–Q) plots were used to inspect the inflation of the test statistics due to any possible systematic bias or error, considering the p-value distribution under the null hypothesis of ‘no association’ (see Supplementary Figure S1). Furthermore, a genomic control method was used to adjust and remove any possible inflation in the test statistics by setting λ to 1 [40]. SNP effects were visualized as −log10 (*p*-value) and are represented on Manhattan plots of each trait, with consideration being given to the relevant chromosome. Two significant thresholds were applied to the SNPs to identify genome- and chromosome-wide significance based on *p*-values. To avoid increased false positives imposed by multiple testing, Bonferroni correction was utilized to assign both genome- and chromosome-wide significance thresholds, where the representative significance level of 0.05 was first divided by the total number of SNPs which passed QC for genome-wide significance. Later, the representative significance level was divided by the average number of SNPs per chromosome to assess for chromosome-wide significance. Thus, the obtained genome-wide significance threshold was 1.236 × 10^−6^ and the chromosome-wide was 3.214 × 10^−5^, respectively leading to 5.90 and 4.49 on the −log10 (*p*-value) scale of the Manhattan plots.

### 2.5. Functional Annotation of Candidate Genes

Genomic positions and nearby genes related to associated SNPs were retrieved from the Oar_v4.0 genome assembly on NCBI Genome Data Viewer [41]. Genes that directly contained significant SNPs were suggested as candidates. However, when the SNP was not within a described gene, the area of the chromosome covering ±250 Kbp from the identified SNP was scanned for the nearest candidate gene with a reasonable explanation. Identified genes were functionally enriched to recover biological information and KEGG (Kyoto Encyclopedia of Genes and Genomes) pathways involved by using The Database for Annotation, Visualization, and Integrated Discovery (DAVID) Bioinformatics Resources 2021 [42,43]. Where the sheep genome suffers from the lack of annotation, the orthology among species was exploited to annotate relevant genes from other species such as cattle, mice, and humans. The biological processes of the identified genes were given with their Gene Ontology (GO) terms and can be further detailed on QuickGO by EMBL’s European Bioinformatics Institute [44]. Finally, the animal QTL Database was scanned to identify whether detected SNPs in this study were previously associated with any growth-, conformation-, and body-type traits [20].

## 3. Results

### 3.1. Phenotypic Characteristics

A total of 17 different traits, including birth weight (**BW**), weaning weight (**WW**), pre-weaning average daily gain (**preADG**), 180 days live weight (**180DW**), post-weaning average daily gain (**postADG**), six months average daily gain (**sixADG**), body condition score (**BCS**), tail size (**TS**), rear legs rear view (**RLRV**), rear legs side view (**RLSV**), rear legs width (**RLW**), rear legs foot angle (**RLFA**), gigot roundness rear view (**GRRV**), gigot roundness side view (**GRSV**), rump width (**RW**), body length (**BL**), and chest width (**CW**) were evaluated for genetic parameter estimations and GWA analysis. Outliers were accordantly removed following linear model fitting. Sex was not significant for any of the measured traits. Birth type was used as an adjustment for the pre-weaning growth traits, while traits recorded after weaning included finishing as an adjustment factor preceding GWASs. Following quality control, the remaining observations for each trait and its relevant descriptive statistics are given in Supplementary Table S1.

### 3.2. Genetic Parameters

Genomic heritabilities as well as genetic and phenotypic correlations among the traits are present in Supplementary Table S2. Overall, moderate heritability estimates were found for growth traits ranging from 0.29 (preADG) to 0.52 (180DW). Contrasting this, the linear type traits had heritabilities distributed within a wider range, between 0.07 (BL) and 0.52 (RLW). In general, positive and high genetic correlations were observed among growth and linear type traits, which supports the idea of using linear type traits as an indicator for growth. A substantial proportion of the genetic correlations among the relevant traits were almost one, indicating a near-perfect linear relationship among the traits. Interestingly, RLW and RLFA had a moderate negative or almost no correlation with some of the traits analyzed. Genetic correlation among the BL and GRRV did not converge to a meaningful number, possibly due to the low genetic variance estimates of the traits. Therefore, these traits were excluded from the results. Finally, phenotypic correlations were observed to be within a low to moderate range among the traits, with the exception of RLW and RLFA, which showed no correlation with most of the traits the dataset contained.

### 3.3. Genome-Wide Association Studies

**Figure 1:**
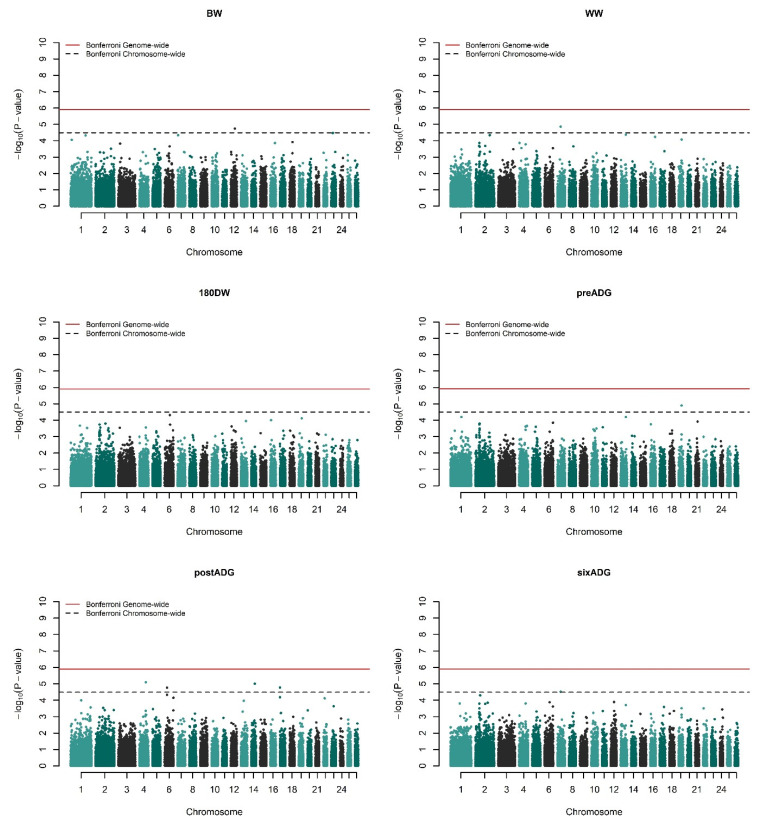
Manhattan plots of growth traits in Akkaraman sheep. −log10 (*p*-values) of the SNPs are plotted against their relevant chromosomes. Horizontal solid red and dashed black lines, respectively, represent genome-wide (5.90) and chromosome-wide significance thresholds (4.49).

**Figure 2:**
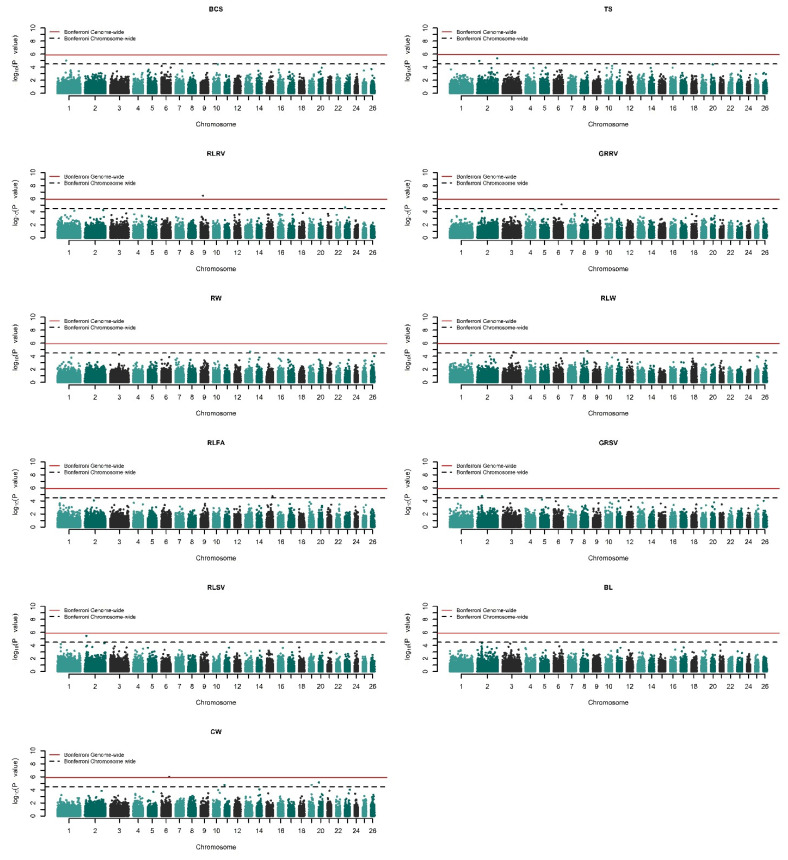
Manhattan plots of the linear type traits in Akkaraman sheep. −log10 (*p*-values) of the SNPs are plotted against their relevant chromosomes. Horizontal solid red and dashed black lines, respectively, represent genome-wide (5.90) and chromosome-wide significance thresholds (4.49).

Genome-wide association studies for 17 growth and type traits (see Supplementary Table S1) were undertaken via univariate mixed model analysis by recursively fitting 40,439 SNPs one at a time and building a genomic relationship matrix. The significant results are presented in Table 1 for growth traits and Table 2 for linear type traits. Q–Q plots of the results (Supplementary Figure S1) show reasonable patterns of associations and no proper sign of inflation. All traits were forced to have a lambda (λ) of approximately 1 by correcting the *p*-values with the genomic control. The corrected *p*-values of the SNPs were visualized with Manhattan plots present in Figure 1 and Figure 2, where the values were converted to −log10 (*p*-value). In total, 20 different SNPs were found to be genome- or chromosome-wide associated with at least one trait at various p-levels. Curiously, three SNPs showed multiple associations among the analyzed traits (see Table 1 and Table 2).

There was a total of seven distinct SNPs that were found to exceed the chromosome-wide significance threshold for analyzed growth traits on *O. aries* chromosomes (OAR) 4, 6, 7, 12, 14, 17, and 19. Among these, the SNP named ‘*rs414279727*’ on OAR7 was common between weaning weight (WW) and 180 days average daily gain (180ADG) as outlined in Table 1. For linear type traits, two SNPs on OAR6 and OAR9 exceeded the genome-wide significance threshold while eleven SNPs on OAR1, 2, 3, 8, 13, 15, and 23 reached chromosome-wide significance for the GWASs (Table 2). Specifically, GWASs for chest width (CW) detected the SNP ‘*rs412278842*’ (*p* = 9.972 × 10^−7^) on OAR6 as genome-wide significant, while the same SNP was also found to be chromosome-wide significant for the gigot roundness rear view (GRRV), *p*-value of 7.323 × 10^−6^. The second genome-wide significant SNP, namely *rs424107094* on OAR9, was associated with the rear legs rear view (RLRV) trait with a p-value of 3.510 × 10^−7^. It is worth noting that four different type traits were associated with five SNPs localized on OAR2. More detailed information on the associated SNPs can be found in Table 1 and Table 2.

### 3.4. Gene Annotation

The Oar_v4.0 genome assembly was utilized on NCBI Genome Data Viewer and UCSC Genome Browser to annotate genomic regions of the associated SNPs and to detect the nearby genes. In total, 17 annotated genes and three uncharacterized loci were suggested as candidates underlying the genetic control of measured growth and linear type traits. Among the identified genes, *PRDM2, PTGDR, PTPRG, KCND2, ZNF260, CPE,* and *GRID2* were associated with growth traits, where *PTGDR* was suggested for both WW and sixADG. Candidates for linear type traits include *SCD5, SPIDR, ZNF407, HCN3, TMEM50A, FKBP1A, TLE4, SP1, SLC44A1*, and *MYOM3* genes located on various chromosomes. Furthermore, *SCD5* was associated with both chest width (CW) and gigot roundness rear view (GRRV), and *TLE4* was associated with both rump width (RW) and gigot roundness side view (GRSV). Loci *LOC105606997, LOC105609002*, and *LOC105602367* were identified as candidates for linear type traits. Further information on the associated SNPs, detected genes, and their relative positions are outlined in Table 1 and Table 2.

## 4. Discussion

Growth and linear type traits are central to sheep selection programmes due to their high influence on production, efficiency, and profitability. However, indigenous sheep breeds, such as Akkaraman, strongly suffer from the lack of a comprehensive pedigree, which is fundamental for a traditional selection program. The use of genomic information in breeding decisions helps overcome this problem by maintaining genomic relationships, where these decisions tend to also result in genetic gain [16,18,19]. The lack of a comprehensive annotation throughout the sheep genome has hampered genome-based selection methods and the understanding of complex traits until recently. With the advent of genomic technologies and the availability of highly annotated sheep reference genomes, it is now possible to use genome-wide distributed markers to understand the genetic structure of the economically important traits in sheep, such as growth and linear type traits. With this purpose, we estimated the heritability of 17 growth and linear type traits based on a genomic relationship matrix. Pairwise genetic and phenotypic correlations were used to understand the nature of the phenotypic variation and the interplay between the 17 growth and linear type traits. Subsequently, we performed a series of GWASs for 17 growth and linear type traits using randomly selected lambs from different herds and revealed two genome-wide and eighteen chromosome-wide associated SNPs with proposed candidate genes.

Heritability estimates of growth traits ranged from 0.29 (preADG) to 0.52 (180DW). In contrast, linear type traits had heritabilities between 0.07 (BL) and 0.52 (RLW). Both trait groups showed similar trends compared to those estimated in Suffolk except for body length, which was calculated as having almost zero heritability in our study [9]. Similarly, estimates for rump width, rear legs rear view, and feet angle were only slightly higher than those obtained from the Spanish Churra breed [5]. While heritabilities for growth traits mirrored what was found in the Churra and Suffolk breeds, computed estimates obtained for growth traits were significantly higher than those determined by Safari et al. and Behrem [13,15]. It is widely known that heritability estimates are crucial elements of an efficient selection index construction process and a prediction of selection response, which emphasizes the importance of accurate estimations for a breeding program. The fact that moderate heritability estimates (Supplementary Table S2), which are consistent with prior results, were found for growth and most linear type traits observed implies a strong potential for faster genetic improvement with a properly designed selection program. Unlike the low genetic and phenotypic correlations found in Bleu de Maine, Texel, and Suffolk breeds, we obtained high correlations within the Akkaraman breed [9]. The support of our results comes from the relatively similar ranges found for growth traits proposed by [15]. Although negative moderate genetic correlations and again negative low phenotypic correlations between BW/WW and BW/preADG were observed previously in Merino sheep, the results of our study suggest an opposite trend for the traits of interest in Akkaraman sheep, with significantly higher positive correlations [13]. Since genetic correlations between traits may be pleiotropic in nature, linkage, and the interplay between background genetic mechanisms are important to reveal and account for while designing a breeding program. Otherwise, targeted selection responses may not be achieved, where one may simultaneously improve certain traits while deteriorating others [45]. The overall high and positive genetic/phenotypic correlations between growth and linear type traits in our study suggest that certain traits may be used as indicators of the others for early and easy breeding decisions; however, negative correlations should be taken into account for the faster genetic improvement of growth.

One of the most salient manifestations of our study is the significant SNPs discovered to be common among the growth and linear type traits, clearly indicating pleiotropy behind the molecular mechanisms of these traits. Given that there are considerably high genetic correlations between WW-sixADG (0.96 ± 0.01), RW-GRSV (0.96 + 0.03), and GRRV-CW (0.96 + 0.03), the common significant SNPs may be explained by genetic interplay. A total of 20 individual SNPs were associated with the analyzed traits at various levels, where three SNPs *rs414279727*, *rs427724890*, and *rs412278842* were commonly found between the abovementioned traits. Further details regarding associated SNPs, *p*-values, candidate genes, and their relative distance are given in Table 1 and Table 2, respectively.

Analyses of associated growth traits led to the identification of seven candidate genes on OAR4, 6, 7, 12, 14, 17, and 19, of which four of the associated SNPs were intron variants of the candidate genes (Table 1). It is important to remember that statistical association is not proof of causality. That also means that some of the significant SNPs might well be in strong linkage disequilibrium with causal mutations that are not represented on the SNP arrays. On the other hand, those SNPs that are within introns also lay the foundations for speculating their potential role in alternative splicing and other gene/protein expression alteration mechanisms. In fact, mounting evidence suggests that introns have expression regulatory roles due to various other mechanisms alongside alternative splicing, which might partially explain the abundance of significant SNPs that are intron variants in this study [46]. Among these candidates, four genes were proposed for postADG and one gene, namely *PTGDR*, was common between WW and sixADG. The *prostaglandin D2 receptor* (*PTGDR*) on OAR7 is a G-protein coupled membrane receptor that functions as a transmembrane signaling receptor (GO: 0004871) and an eicosanoid receptor (GO: 0004953). Interestingly, the suggested gene was previously found in a selection signature study conducted on thin- and fat-tailed sheep [47]. Additionally, *PTGDR* has also been associated with hot carcass weight and body depth in various cattle breeds [48,49]. On the other hand, SNP *rs423706103* on OAR19 encompasses the intronic variant of the *protein tyrosine phosphatase receptor type G* (*PTPRG*) gene. *PTPRG* carries phosphoric ester hydrolase activity (GO: 0042578) and was associated with preADG in our study. Support for this gene as a functional candidate comes from [50], which maintained similar results. On the other hand, postADG was associated with SNP *rs407771300* on OAR4, which is present at an intronic variant of *potassium voltage-gated channel subfamily D member 2 (KCND2)*. As a transmembrane protein, *KCND2* is responsible for protein complex assembly (GO: 0065003), ion transport (GO: 0006811), and the establishment of localization (GO: 0051234). Further, KEGG analysis also suggests that it mediates serotonergic synapses. Concordantly, another SNP within *KCND2* was formerly associated with withers height in sheep [51]. Lastly, it is worth mentioning SNPs *rs404771550* and *rs399086810*, which are associated with postADG and RLRV, respectively, as they identified candidate genes that were zinc finger proteins. Specifically, *zinc finger protein 260* (*ZNF260*) was found approximately 100 bp downstream of SNP *rs404771550* and an intron variant of *ZNF407* was identified at SNP *rs399086810*. Zinc-finger proteins (ZNFs) have long been known to be involved in the growth and development of various species [52]. Additionally, various members of ZNFs, *ZNF395*, and *ZNF641*, have already been associated with growth traits in sheep [24,53]. Therefore, our study further validates these previous findings as various ZNFs have already proved their worth for genetic improvement regarding sheep growth.

The two genome-wide associated SNPs in our study, namely *rs412278842* on OAR6 and *rs424107094* on OAR9, were discovered as a result of GWASs for CW and RLRV, respectively. The former SNP was also found to be associated with GRRV on a chromosome-wide level. This SNP is located approximately 100 Kb upstream of the *stearoyl-CoA desaturase 5 (SCD5)* gene which has a critical role in the fatty acid metabolic process (GO: 0006631) and the lipid biosynthetic process (GO: 0008610). KEGG analysis further validated *SCD5*’s role in fatty acid metabolism and the biosynthesis of unsaturated fatty acids but added the AMPK signaling pathway. A recent study implemented whole-genome tests for various economically important traits in sheep and suggested *SCD5* as a strong candidate for body size [54]. Given that *SCD5* was previously proposed to partake in the body size development of grass-fed Brangus steers [55], there is support for this gene as a strong candidate involved in sheep growth. The second highly and genome-wide significant SNP was found to be associated with RLRV and lies within the gene *scaffold protein involved in DNA repair* (*SPIDR*) of OAR9 as an intron variant. *SPIDR* is a nucleoplasm protein with key functions such as the regulation of DNA recombination (GO: 0000018), DNA repair (GO: 0006281), regulation of macromolecule metabolic processes (GO: 0010604), response to hydroxyurea (GO: 0072710), and response to stress (GO: 0080134). Apart from the discussed genome-wide significant SNPs, one SNP named *rs427724890* on OAR2 showed a chromosome-wide association with both the RW and GRSV type traits. The associated SNP was located only 1–2 Kb upstream from the gene which encodes *Transducin-like enhancer protein 4* (*TLE4*), a transcriptional co-repressor. Functional annotation within KEGG pathways showed that it is involved in Wnt and Notch signaling pathways. As a prominent regulator of the canonical Wnt signaling pathway (GO: 0016055) and cellular biosynthetic process (GO: 0031326), TLE4 was recently suggested to regulate muscle satellite cell quiescence and muscle differentiation by repressing PAX7-mediated *MYF5* transcriptional activation in mice [56].

It is worth noting that an abundance of SNPs on OAR2 was associated with various linear type traits. In total, five SNPs on OAR2 were associated with four different linear type traits, including RW, TS, GRSV, and RLSV. As indicated by Table 2, the candidate genes associated with tail size (TS) included *TLE4*, *transmembrane protein 50A (TMEM50A)*, and *LOC105606997*, where *TLE4* was mentioned above as it was also found to be associated with RW and GRSV traits. *Myomesin 3 (MYOM3)* and *solute carrier family 44 member 1 (SLC44A1)* were candidate genes identified for linear type trait RLSV. Various TMEM’s, including *TMEM8B* on OAR2, have been suggested to have relationships with body weight and body length in sheep [51,57]. Selection signature scans conducted on worldwide sheep populations by Fariello et al. (2014) also suggested *TMEM50A* as a candidate for further investigations [58]. On the other hand, MYOM3 protein is a member of the structural proteins found within the M-band of striated muscle sarcomeres (GO: 0005515), which specifically have actin filament binding activity (GO: 0051015). Considering the previously associated regions on OAR2 regarding sheep growth and conformation [10,12,57,59], it would be fair to suggest that OAR2 plays an important role in the phenotypic determination of growth and type traits. Finally, SNP *rs405451961* is associated with BCS within our current study and is located within the 10th exon of the *hyperpolarization-activated cyclic nucleotide gated potassium channel 3* (*HCN3*) on OAR1. HCN3 is suggested to regulate biological quality (GO: 0065008), measured by animal size, mass, shape, and color, and to be involved in the regulation of membrane potential (GO: 0042391). KEGG analysis showed that HCN3 is also involved in GnRH secretion.

The functional enrichment of the identified genes through KEGG pathway analysis determined that the *SP1* gene is involved in endocrine resistance, cortisol synthesis, parathyroid hormone synthesis, and TGF-β signaling. Furthermore, the *PRDM2* gene was suggested to play a role in various metabolic processes, such as lysine degradation. Finally, the *CPE* gene was suggested to influence Type I diabetes mellitus whereas *GRID2* and *PTGDR* are responsible for neuroactive ligand-receptor interaction. All in all, we have found 20 associated SNPs with 20 suggested candidates, where their biological importance in growth and development is well observed. The discovery of common SNPs between the traits further supports the relatively high genetic correlations among them and indicates a shared genetic background. Although sheep genomics has recently had breakthrough improvements, for instance the increase in genome assembly publications and the number of high-density SNP arrays, sheep growth, and type traits have only undergone a few studies. Therefore, our results are expected to significantly contribute to the potential knowledge of the genetic background of growth and conformation in sheep, which are central to the economic revenue in sheep production systems.

## 5. Conclusions

In this study, the genetic architecture of 17 growth and linear type traits were investigated in Akkaraman sheep. Correspondingly, moderate heritabilities as well as high and positive genetic/phenotypic correlations were observed. This indicates that 1) there is enough genetic variance to be exploited for faster genetic improvement of the focused traits and 2) observed genetic and phenotypic correlations among the traits can be used to define the potential of the traits as indicators for early and easy detection of the breeding male/females. Moreover, two genome-wide and eighteen chromosome-wide significant SNPs were found to be associated with the traits as a result of performed GWA analyses. Accordingly, *PRDM2, PTGDR, PTPRG, KCND2, ZNF260, CPE*, *GRID2, SCD5, SPIDR, ZNF407, HCN3, TMEM50A, FKBP1A, TLE4, SP1, SLC44A1*, and *MYOM3* were proposed as the genes bearing putative QTLs for growth and linear type traits. Certain genes in our study were found to be common between the measured traits, which suggests that an interplay between the genetic backgrounds of these traits exists. Additionally, the fact that four of the suggested genes were found to be located on OAR2 further emphasizes the importance of the chromosome for growth and morphological structure in sheep. Considering the wide influence of these traits on the other traits of economic importance, these results carry important implications regarding marker-assisted selection programs as well as understanding the genetic architecture of growth and linear type traits and pointing out loci for targeted genome-editing/gene-knockout studies.

To our knowledge, this is among the first studies concerning genetic parameters and the genetic background of linear type traits and growth in an indigenous sheep breed. Future genomic studies such as those targeting parasite resistance, disease susceptibility, and fleece characteristics should be considered. Together, these data could be used to design a comprehensive breeding program for sheep, specifically for Akkaraman sheep. In turn, the efficiency, profitability, and sustainability of sheep production systems will benefit. Nevertheless, considering the low number of discovered QTLs, it is extremely important to implement more discovery studies with an increased number of animals to shed a light on the complex molecular mechanisms behind growth and type traits.

## Figures and Tables

**Table 1:** Significant SNPs for growth traits.

Trait	SNP Name	OAR	Oar_v4.0 Position (bp)	*p*-Value	Sig.	MAF	Candidate Gene	Distance
postADG	*rs407771300*	4	85,034,136	7.974 × 10^−6^	CW	0.486	*KCND2*	Intron variant
postADG	*rs404771550*	14	46,046,471	9.876 × 10^−6^	CW	0.253	*ZNF260*	~100 bp upstream
preADG	*rs423706103*	19	39,758,712	1.270 × 10^−5^	CW	0.276	*PTPRG*	Intron variant
WW	***rs414279727***	7	42,283,714	1.377 × 10^−5^	CW	0.479	*PTGDR*	~50 Kb downstream
postADG	*rs400100688*	17	448,355	1.677 × 10^−5^	CW	0.121	*CPE*	Intron variant
postADG	*rs429448354*	6	30,967,377	1.701 × 10^−5^	CW	0.424	*GRID2*	Intron variant
BW	*rs414781462*	12	51,905,842	1.793 × 10^−5^	CW	0.262	*PRDM2*	25 Kb upstream
sixADG	***rs414279727***	7	42,283,714	3.031 × 10^−5^	CW	0.479	*PTGDR*	~50 Kb downstream

Notes: OAR = *O. aries* chromosome; Sig.= significance; MAF = Minor Allele Frequency. Bold SNP names indicate those that were found common among traits. Here, BW, WW, preADG, postADG, and sixADG, respectively, stand for birth weight, weaning weight, and pre-weaning average daily gain, post-weaning average daily gain, and six months average daily gain. Regarding the significance column, GW stands for genome-wide and CW for chromosome-wide significant SNPs.

**Table 2:** Significant SNPs for linear type traits.

Trait	SNP Name	OAR	Oar_v4.0 Position (bp)	*p*-Value	Sig.	MAF	Candidate Gene	Distance
RLRV	*rs424107094*	9	32,023,081	3.510 × 10^−7^	**GW**	0.220	*SPIDR*	Intron variant
CW	***rs412278842***	6	97,145,365	9.972 × 10^−7^	**GW**	0.126	*SCD5*	~100 Kb upstream
RLSV	*rs411373597*	2	17,728,536	3.243 × 10^−6^	CW	0.258	*SLC44A1*	~250 bp upstream
TS	*rs417737929*	2	240,680,051	4.327 × 10^−6^	CW	0.050	*TMEM50A*	~100 Kb downstream
GRRV	***rs412278842***	6	97,145,365	7.323 × 10^−6^	CW	0.126	*SCD5*	~100 Kb upstream
BCS	*rs405451961*	1	103,920,255	9.628 × 10^−6^	CW	0.399	*HCN3*	Exon variant
TS	*rs404544718*	2	25,145,453	1.135 × 10^−5^	CW	0.347	*LOC105606997*	~15 Kb upstream
RLW	*rs417934245*	8	81,117,831	1.668 × 10^−5^	CW	0.356	*LOC105609002*	~90 Kb downstream
GRSV	***rs427724890***	2	56,856,646	1.769 × 10^−5^	CW	0.120	*TLE4*	~1–2 Kb upstream
RW	*AX-123238082*	13	58,972,752	1.897 × 10^−5^	CW	0.326	*FKBP1A*	~15 Kb
RLFA	*rs398187690*	15	73,532,306	1.912 × 10^−5^	CW	0.156	*LOC105602367*	~90 Kb
RLW	*rs410532183*	3	132,823,805	2.189 × 10^−5^	CW	0.169	*SP1*	~ 1Kb upstream
RLRV	*rs399086810*	23	3,643,516	2.215 × 10^−5^	CW	0.454	*ZNF407*	Intron variant
RW	***rs427724890***	2	56,856,646	2.762 × 10^−5^	CW	0.120	*TLE4*	~1–2 Kb upstream
RLSV	*rs409929874*	2	241,787,646	3.320 × 10^−5^	CW	0.450	MYOM3	~30 Kb downstream

Notes: OAR = O. arieschromosome; Sig. = significance; MAF = Minor Allele Frequency. Bold SNP names indicate those that were found common among tested traits. Regarding the trait column, CW, RLRV, BCS, TS, GRRV, RW, RLW, RLFA, GRSV, and RLSV are, respectively, acronyms for chest width, rear legs rear view, body condition score, tail size, gigot roundness rear view, rump width, rear legs width, rear legs foot angle, gigot roundness side view, and rear legs side view. Within the significance column, GW stands for genome-wide and CW for chromosome-wide significant SNPs. GW values are also bolded for ease of identification.

## Data Availability

The data presented in this study are available on a reasonable request from the corresponding author.

## Supplementary Materials

The following supporting information can be downloaded at: https://www.mdpi.com/article/10.3390/genes13081414/s1, Supplementary Figure S1: Q-Q plots of GWA analyses; Supplementary Table S1: Descriptive statistics of growth and linear type traits; Supplementary Table S2: Heritability, genetic and phenotypic correlations of growth and linear type traits.
