# Supplementary material for: Genetic Parameters and Genomic Regions Underlying Growth and Linear Type Traits in Akkaraman Sheep
Source: Genes (Basel). 2022 Aug 10;13(8):1414. doi: 10.3390/genes13081414 (PMC9407525; doi:10.3390/genes13081414)
Supplement: Supplementary file 1 [file genes-13-01414-s001.zip › Suplementary Table S1_Descriptive_Statistics_MK_YA_MUC_SW_LP.pdf]

Suplementary Table S1. Descriptive statistics of growth and linear type traits

Trait	N	Min.	Mean	SD	Max
**BW (kg)**	451	2.96	4.59	0.71	6.54
**WW (kg)**	449	14.10	25.71	4.29	36.61
**180DW (kg)**	452	19.33	42.87	7.99	68.05
**preADG**	450	72	234	46	365
**postADG**	456	11	168	71	396
**180ADG**	451	82	212	70	352
**BCS**	473	2.00	3.66	0.98	5.00
**TS**	469	2.00	4.78	1.46	8.00
**RLRV**	470	2	3.68	0.81	5
**GRRV**	471	4	5.35	0.89	7
**RW**	473	3	5.38	1.34	8
**RLW**	473	2	3.39	0.92	5
**RLFA**	473	2	3.39	0.92	5
**GRSV**	472	3	5.39	0.87	7
**RLSV**	469	3	4.41	0.66	5
**BL**	473	4	4.91	0.82	7
**CW**	470	3	5.34	1.02	8

Notes: **N**= Number of observations; **SD**= Standard deviation; **BW**= Birth weight; **WW**= Weaning weight; **180DW**= 180 days adjusted weight; **preADG**= pre-weaning average daily gain; **postADG**=post-weaning average daily gain; **180ADG**= 180 days average daily gain; **BCS**= Body condition score; **TS**= Tail size; **RLRV**=Rear legs (rear view); **GRRV**= Gigot roundness (rear view); **RW**= Rump width; **RLW**= Rear legs width; **RLFA**= Rear legs foot angle; **GRSV**= Gigot roundness (side view); **RLSV**= Rear legs (side view); **BL**= Body length; **CW**= Chest width
