# Supplementary material for: Genetic Parameters and Genomic Regions Underlying Growth and Linear Type Traits in Akkaraman Sheep
Source: Genes (Basel). 2022 Aug 10;13(8):1414. doi: 10.3390/genes13081414 (PMC9407525; doi:10.3390/genes13081414)
Supplement: Supplementary file 1 [file genes-13-01414-s001.zip › Supplementary Table S2_Genetic_Parameters_MK_YA_MUC_SW_LP.pdf]

Supplementary Table S2. Heritability, genetic and phenotypic correlations of growth and linear type traits.

Trait	BW	WW	180DW	preADG	postADG	sixADG	BCS	TS	RLRV	GRRV	RW	RLW	RLFA	GRSV	RLSV	BL	CW
**BW**	**.39(.12)**	.32(.04)	.34(.04)	.16(.04)	.30(.04)	.26(.04)	.21(.04)	.28(.04)	.10(.04)	.19(.04)	.13(.04)	-.11(.04)	.03(.04)	.17(.04)	.09(.04)	.22(.04)	.14(.04)
**WW**	.88(.13)	**.38(.13)**	.85(.04)	.98(.03)	.34(.03)	.84(.00)	.30(.04)	.60(.04)	.18(.04)	.50(.04)	.42(.04)	-.03(.04)	-.10(.04)	.50(.04)	.08(.04)	.53(.04)	.43(.04)
**180DW**	.96(.13)	. 92(.04)	**.52(.13)**	.83(.02)	.75(.03)	.99(.00)	.38(.04)	.67(.03)	.24(.04)	.56(.04)	.48(.04)	.05(.04)	.00(.04)	.56(.04)	.08(.04)	.66(.04)	.53(.04)
**preADG**	.60(.19)	.99(.01)	.95(.01)	**.29(.12)**	.31(.04)	.84(.02)	.29(.04)	.58(.04)	.17(.04)	.49(.04)	.42(.04)	-.01(.04)	-.10(.04)	.51(.04)	.06(.04)	.52(.04)	.43(.04)
**postADG**	.71(.14)	.69(.17)	.96(.03)	.77(.14)	**.51(.13)**	.75(.04)	.29(.04)	.47(.04)	.21(.04)	.38(.04)	.33(.04)	.08(.04)	.08(.04)	.39(.04)	.08(.04)	.50(.04)	.39(.04)
**sixADG**	.83(.16)	.96(.01)	1.00(.00)	.96(.01)	.97(.02)	**.51(.13)**	.38(.04)	.66(.03)	.24(.04)	.56(.04)	.49(.04)	.06(.04)	.00(.04)	.57(.04)	.08(.04)	.66(.04)	.53(.04)
**BCS**	.62(.18)	.72(.13)	.94(.12)	.74(.14)	.91(.21)	.97(.12)	**.32(.12)**	.45(.04)	.06(.04)	.47(.04)	.45(.04)	.01(.04)	.01(.04)	.35(.04)	.00(.04)	.34(.04)	.46(.04)
**TS**	.75(.15)	.91(.05)	.91(.04)	.90(.05)	.81(.09)	.91(.04)	.80(.08)	**.50(.13)**	.15(.04)	.71(.04)	.67(.04)	-.02(.04)	-.05(.04)	.60(.04)	.06(.04)	.63(.04)	.63(.04)
**RLRV**	.30(.17)	.49(.15)	.64(.16)	.51(.17)	.58(.17)	.65(.16)	.21(.19)	.41(.16)	**.26(.11)**	.09(.04)	.03(.04)	.17(0.04)	.13(.04)	.04(.04)	.12(.04)	.15(.04)	.08(.04)
**GRRV**	.59(.18)	.89(.07)	.91(.06)	.90(.07)	.86(.12)	.92(.06)	.96(.07)	.91(.03)	.33(.19)	**.30(.12)**	.72(.04)	.03(.04)	-.02(.04)	.65(.03)	.04(.04)	.62(.04)	.69(.03)
**RW**	.41(.17)	.84(.09)	.82(.07)	.88(.09)	.71(.12)	.83(.07)	.85(.08)	.87(.04)	.12(.21)	.83(.14)	**.25(.11)**	.10(.04)	-.03(.04)	.67(.04)	.02(.03)	.60(.03)	.71(.03)
**RLW**	-.25(.14)	-.02(.12)	.24(.09)	-.03(.14)	.31(.09)	.26(.10)	-.66(.25)	.04(.12)	.44(.12)	.16(.14)	.32(.15)	**.52(.13)**	.03(.04)	.04(.04)	.01(.04)	.19(.04)	.04(.04)
**RLFA**	.57(.51)	-.30(.20)	.14(.29)	-.33(.21)	.39(.32)	.14(.29)	.14(.36)	-.19(.26)	.65(.33)	-.04(.35)	-.08(.38)	.11(.28)	**.39(.12)**	-.09(.04)	.27(.04)	.00(.04)	-.02(.04)
**GRSV**	.46(.17)	.88(.07)	.87(.06)	.91(.07)	.81(.11)	.88(.06)	.83(.12)	.88(.05)	.14(.20)	.97(.03)	.96(.03)	.11(.15)	-.31(.28)	**.20(.10)**	.10(.04)	.67(.04)	.68(.03)
**RLSV**	.36(.28)	.31(.28)	.32(.28)	.32(.27)	.36(.32)	.29(.28)	-.09(.25)	.22(.24)	.78(.38)	.03(.27)	-.09(.31)	.00(.22)	.96(.14)	.31(.26)	**.16(.09)**	.07(.04)	.07(.04)
**BL**	.90(.40)	.97(.05)	.92(.04)	.98(.06)	.88(.07)	.93(.04)	.94(.13)	.92(.04)	.68(.30)	NA	.99(.04)	.59(.16)	.21(.62)	1.00(.25)	.25(.42)	**.07(.07)**	.62(.04)
**CW**	.43(.17)	.86(.09)	.81(.06)	.90(.09)	.71(.09)	.82(.07)	.79(.07)	.83(.05)	.31(.20)	.96(.03)	.92(.11)	.15(.15)	.06(.41)	.97(.03)	.23(.26)	1.00(.21)	**.30(.11)**

Notes: Diagonal values represent the genomic heritability of the overlapping trait where phenotypic correlations are above the diagonal and genetic correlations below the diagonal.
NA= Could not be reliably estimated.
