# Supplementary figures and images for: Genetic Parameters and Genomic Regions Underlying Growth and Linear Type Traits in Akkaraman Sheep
Source: Genes (Basel). 2022 Aug 10;13(8):1414. doi: 10.3390/genes13081414 (PMC9407525; doi:10.3390/genes13081414)

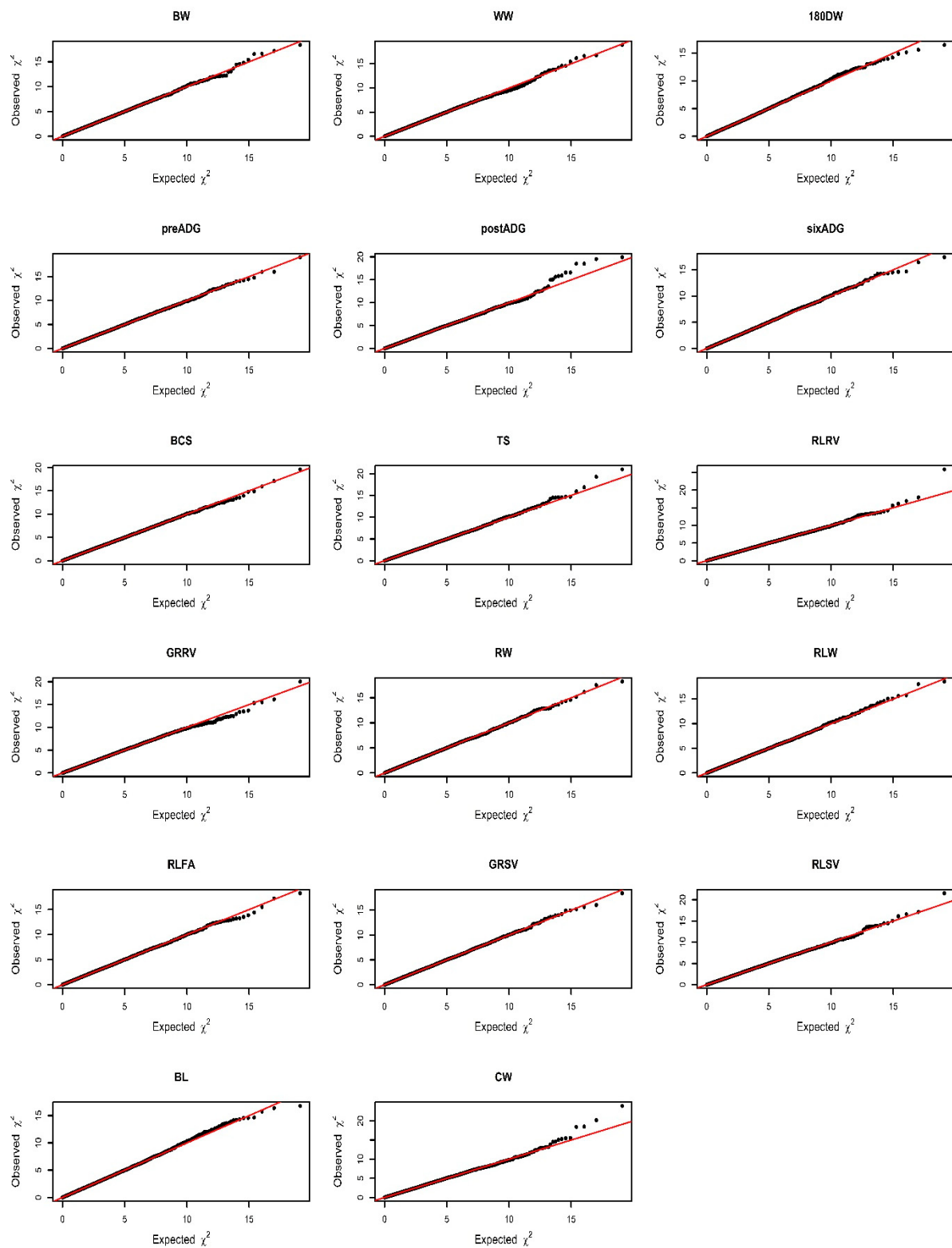

**Supplementary Figure S1. Q-Q plots of GWA analyses**

Supplement: Supplementary file 1 [file genes-13-01414-s001.zip › Supplementary Figure S1.pdf]
